# Evaluation of Marginal Fit of CAD/CAM Ceramic Crowns and Scanning Time Using Different Intraoral Scanning Systems

**DOI:** 10.3390/jfb15120359

**Published:** 2024-11-26

**Authors:** Leandro Maruki Pereira, Bárbara Inácio de Melo, Alex Antônio Maciel Oliveira, Gustavo Mendonça, Luís Henrique Araújo Raposo, Marcel Santana Prudente, Flávio Domingues das Neves

**Affiliations:** 1 Department of Occlusion, Fixed Prosthodontics and Dental Materials, School of Dentistry, Federal University of Uberlandia, Uberlandia 38405-320, Minas Gerais, Brazil; leandromaruki@gmail.com (L.M.P.); barbarainaciodemelo@gmail.com (B.I.d.M.); raposo@ufu.br (L.H.A.R.); flaviodominguesneves@gmail.com (F.D.d.N.); 2 Private Practice, Odontocenter, Pompéu 35640-000, Minas Gerais, Brazil; alexamaciel@hotmail.com; 3 Department of General Practice, Virginia Commonwealth University School of Dentistry, Richmond, VA 2329-05668, USA; mendoncag@vcu.edu

**Keywords:** CAD/CAM, ceramics, crown, marginal fit

## Abstract

This study aimed to evaluate the scanning time and marginal fit of CAD/CAM crowns fabricated using different intraoral scanning systems (IOS) (O1—Omnicam 1.0, O2—Omnicam 2.0, PS—Primescan). A standardized, 3D-printed composite resin die with a full-crown tooth preparation was scanned ten times with each IOS, and the scanning time was recorded. Subsequently, lithium disilicate ceramic crowns were designed and milled. The crowns were seated in the die and scanned using micro-computed tomography to assess the marginal fit. Fifty-two measurements were performed for each crown. Data were assessed for homogeneity, and one-way analysis of variance and the Tukey HSD test were performed (α = 0.05). For the analysis of vertical fit categories, the chi-square (Fisher’s exact) test (α = 0.05) was used. The mean vertical fit values were: O1—46.7 ± 16.4 μm, O2—33.8 ± 21.4 μm (*p* = 0.041), and PS—12.3 ± 6.6 μm (*p* < 0.001). The vertical fit values were further categorized by percentage and representative specimens were scanned with electron microscopy to evaluate adaptation. The mean scanning times were: O1—37.4 ± 3.1 s; O2—34.8 ± 2.7 s; and PS—27.8 ± 1.9 s. Significant differences were observed in the scanning times and marginal fit values of the CAD/CAM ceramic crowns across the different IOS systems, with PS demonstrating the best results. Improvements in IOS hardware and software significantly influence these outcomes.

## 1. Introduction

The increasing emphasis on aesthetics in dentistry has driven the widespread adoption of all-ceramic restorations [1]. The precise fabrication of ceramic crowns is crucial for the long-term success of treatment [2]. Imprecise impressions may result in restorations with inadequate marginal fit [2,3]. Digital impression methods offer several advantages, including real-time visualization of three-dimensional (3D) models, correction of the scanning procedure during the procedure [2], time optimization, and the possibility of chair-side production for indirect restorations using computer-assisted design/computer-aided manufacturing (CAD/CAM) [4,5,6,7,8]. This procedure is performed using intraoral scanning systems (IOSs), which have been further enhanced with software and hardware updates to improve the fit of restorations [4].

The CEREC intraoral scanning system has undergone substantial updates over the years, particularly with respect to its image capture technology [9,10]. These updates minimize errors and reduce marginal discrepancies [9,10]. The Omnicam system generates a 3D model, using a video sequence with active triangulation and strip-light projection technology [11,12]. Active techniques project light from the camera onto the object, minimizing dependence on the real texture and color of tissues for reconstruction [13,14]. In this approach, a luminous point is projected onto an object and the distance to the object is calculated by triangulation. The new Primescan system utilizes a video sequence and confocal microscopy technology [15]. This technique captures focused and defocused images at selected depths [14] and employs structured light [5]. Additionally, the Primescan has a larger field of view than the Omnicam, making the Primescan faster and more accurate [5,16].

The first CEREC chair-side system was developed in 1984. Since then, this system has significantly advanced intraoral scanning technology [10]. Bluecam was initially introduced as a 3D system employing blue-light image capture technology, followed by the video-based Omnicam and Primescan scanners [10]. Cameras with newer technologies are expected to provide similar or improved results to existing technologies in terms of scanning time, fit, and accuracy of restorations [10,16]. Marginal fit is an important aspect of the clinical longevity and success of indirect restorations. However, inadequate crown adaptation can result in microleakage, cement solubility, plaque accumulation, secondary caries, periodontal tissue inflammation, and potential endodontic complications [8,9,17,18,19]. Several factors, such as the software and hardware used [3], operator experience [20], and tooth preparation design have been shown to influence the marginal fit of crowns [21,22]. There is currently no consensus on the clinically acceptable marginal discrepancies for indirect restorations [9,10,23,24,25]. Clinically, a fit is considered acceptable when the margin interface cannot be detected with an explorer [23]. Some studies suggest that a marginal fit below 120 µm is acceptable [24,26,27], while others recommend values below 100 µm [28,29]. Additionally, some investigations propose that the acceptable fit should be under 75 µm [30]. Ideally, the clinically perfect marginal fit for cemented restorations ranges from 25 µm to 40 µm [9,31], although achieving these values remains a challenge [32].

Comparing marginal fit data from various studies is challenging due to the use of different assessment methods [20,27]. Some methods are destructive, such as sectioning crowns with a diamond disc and measuring the marginal gap with stereomicroscopy, or with scanning electron microscopy [33,34,35]. However, non-destructive methods are also available, such as 3D superimposition techniques, cement thickness measurements with polyvinylsiloxane (PVS) paste, and micro-computed tomography (micro-CT) analysis [9,32,36,37,38,39,40]. Micro-CT allows detailed 3D evaluation of marginal fit with micron-level accuracy at different sites and orientations [38,41]. While this method offers qualitative and quantitative analyses of the vertical, horizontal, and internal fit of crowns along the coronal and sagittal axes, it presents challenges, including high acquisition costs, extended scanning times, and complex data analysis [10,38].

Previous studies reported marginal crown adaptation values for the Omnicam scanner ranging between 88.24 μm [42] and 149.4 μm [10]. The Omnicam employs triangulation-based image capture technology [14], but this method has limitations that may impact the accuracy of restorations [43]. In contrast, the Bluecam scanner, which uses confocal technology [14], has achieved results ranging from 29.5 μm [10] to 63.75 μm [42], demonstrating superior performance compared to the Omnicam. However, despite advancements that promise improved results, the practical application of these updates may vary [25,43]. The new Primescan scanner also incorporates confocal technology [9], refining the Bluecam model with video-capture capabilities. Therefore, the current study aimed to investigate the differences between these two intraoral optical scanning technologies, focusing on the scanning time and marginal fit of fabricated crowns. The null hypothesis was that the scanning time and marginal fit of ceramic crowns would not differ between the IOSs tested.

## 2. Materials and Methods

### 2.1. Manufacturing the Crowns

**Figure 1:**
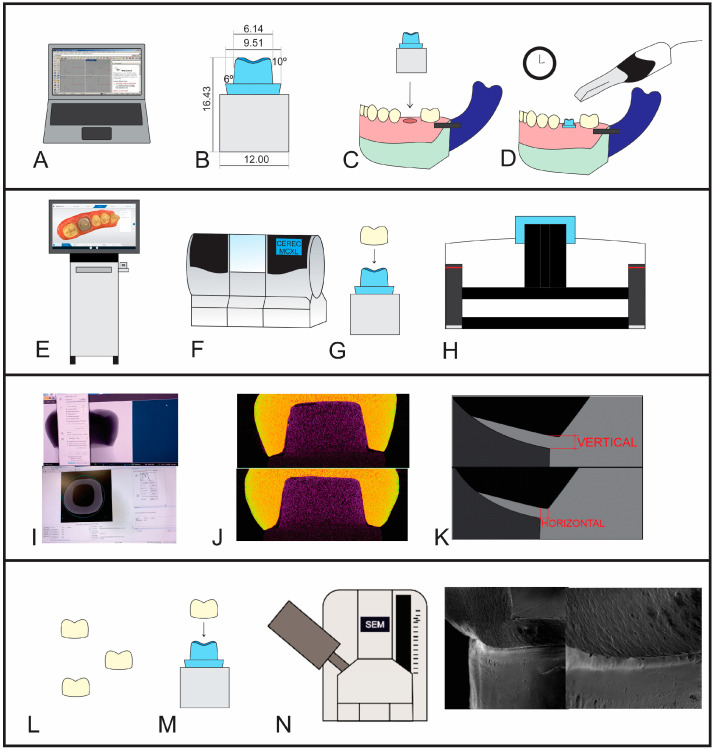
Methodological steps: (**A**) design of the preparation, (**B**) preparation details, (**C**) fixation of the preparation in a typodont model, (**D**) scanning (*n* = 10) and time recording, (**E**) design of the crown, (**F**) crown milling, (**G**) crown seated on the die, (**H**) micro-CT scanning, (**I**) reconstruction of the scans, (**J**) image selection, (**K**) marginal fit measurements, (**L**) representative crowns, (**M**) crown seated on the die, and (**N**) scanning electron microscopy.

A standardized die with a full-crown preparation was designed using 3D modeling [44] in CAD software (Rhinoceros 4.0, McNeel North American, Seattle, WA, USA) and NURBS lines and then printed in photocured resin composite using a 3D printer (Objet Connex350, Stratasys, Eden Prairie, MN, USA) (Figure 1A). The die represented a full-crown preparation of the mandibular left first molar, with rounded axiogingival angles and shoulder termination (Figure 1B). Before the die was fixed on a full-arch typodont model (Figure 1C), the preparation was partially scanned ten times using the following IOS systems: Omnicam 1.0 (Sirona Dental Systems GmbH, Bensheim, Germany), Omnicam 2.0, (Sirona Dental Systems GmbH, Bensheim, Germany), and Primescan (Sirona Dental Systems GmbH, Bensheim, Germany) (Figure 1D), as described in Table 1. The scanning process was automated by software which defined the insertion axis and preparation margin. In addition, for the O1 and O2 systems, the composite resin die was sprayed with a thin layer of opacifier powder (CEREC Optispray Sirona Dental Systems GmbH, Bensheim, Germany) to improve scanning [10]. The scanning time was recorded for each scan performed using the different IOS systems. The sample size was determined considering previous studies (*n* = 10) [9,10].

The Omnicam 1.0 crowns were designed using CEREC v. 4.2.5, while the Omnicam 2.0 and Primescan crowns were designed using CEREC 3D v. 5.0 software. The luting space was set at 80 μm, according to the manufacturer’s instructions. The margins of the restorations were manually adjusted. After designing the crowns, the ceramic blocks (Figure 1E) were milled in a computer-controlled milling unit (MCXL, CEREC, Sirona Dental GmbH) in the veneer milling mode (Figure 1F). Lithium disilicate (LS2) reinforced glass ceramic CAD/CAM blocks (LOT YB552T, IPS e.max CAD; MT-A2 shade; Ivoclar Vivadent, Schaan, Liechtenstein) were used to produce the crowns in the three IOS systems, as shown in Table 1. No internal adjustments were made, and crystallization of the crowns was performed according to the manufacturer’s instructions, using the same firing program (Program P91, Programat P300, Ivoclar Vivadent, Schaan, Liechtenstein), as shown in Table 2.

### 2.2. Micro-Computed Tomography and Marginal Fit Measurements

Each crown was seated on the standardized die and fixed with a PVS-based material (GC Fit Checker, GC Dental Industrial Corp., Tokyo, Japan) using custom equipment to uniformly apply 20 N of pressure during the material setting (digital pressure) (Figure 1G). The crown-die sets were individually digitized using micro-computed tomography (SkyScan 1272; Bruker microCT, Kontich, Belgium) to obtain images for marginal fit measurements. Micro-CT scans were performed at 100 Kv and 100 μA, with a pixel size of 9.4 μm, Cu filter of 0.11 mm, and resolution of 1632 × 1092 pixels. Selected scanning was performed in rotation steps from 0.6 to 360 degrees, and two frames with random movements of 20 pixels were collected, resulting in a scanning time of 38 min per specimen (Figure 1H).

**Figure 2:**
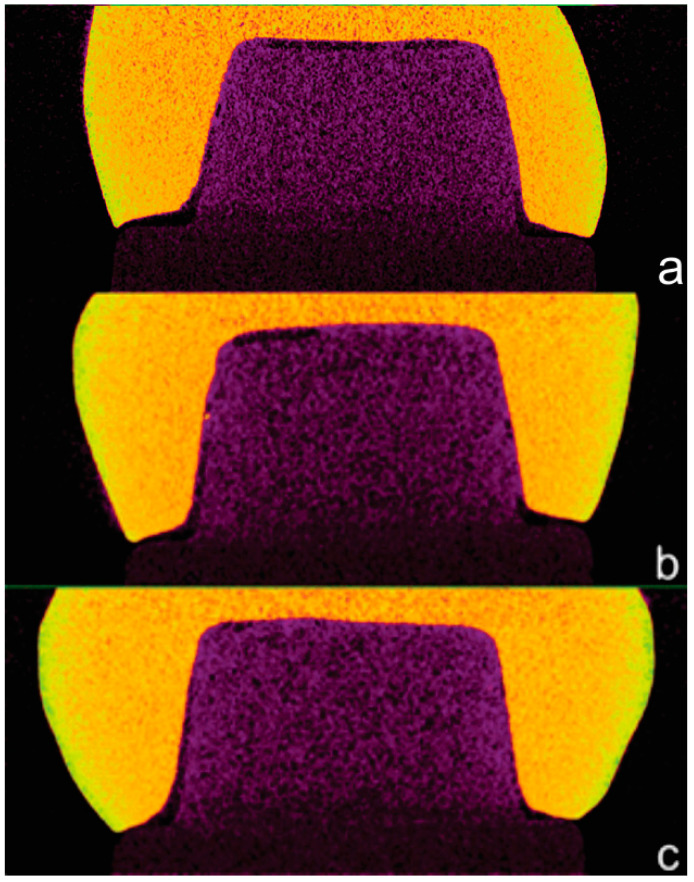
Micro-CT images: (**a**) O1 group, (**b**) O2 group, and (**c**) PS group.

Subsequently, the micro-CT images were reconstructed (Figure 1I), and the existing artifacts were reduced. NRecon software (v. 1.1.8.0., SkyScan; Bruker microCT, Kontich, Belgium) was used with the following parameters: 5% smoothing, 4% ring artifact correction, and 5% beam-hardening correction. Next, Dataviewer software (v. 1.5.0.2; SkyScan; Bruker microCT, Kontich, Belgium) was used to obtain the sagittal and coronal image sets (Figure 1J), and Figure 2 presents examples of the selected images. Subsequently, 13 images were selected for the sagittal and coronal sets, showing the entire specimen length in two different orientations (mesiodistal and buccolingual). The images were chosen from the same spatial division between the first and last images where the cervical margins appear [9].

The marginal fit measurements were made in each of the selected images, with two readings for vertical and horizontal adaptation at 600× magnification, using CTAN processing software (v. 1.12.0.0, SkyScan). Fifty-two measurements were performed per specimen and were equally divided between the mesial buccal, lingual, and buccal surfaces, according to previous investigations [10,23]. These measurements were assigned to their respective surfaces to assess any relationship of the marginal fit per region. The vertical fit was measured parallel to the path of the tooth preparation limit, while the horizontal fit was assessed perpendicular to the path of the tooth preparation limit (Figure 1K). The measurements were performed by three previously calibrated evaluators and the average values of the three assessments were considered (Kappa = 0.80).

### 2.3. Scanning Electron Microscopy Analysis

Representative specimens were selected from each group based on their marginal fit values, which closely aligned with the overall group average (Figure 1L). These specimens were previously assessed using micro-CT (Figure 1M), and then prepared for scanning electron microscopy (SEM) analysis (VEGA 3 LMU, Tescan, Brno, Czech Republic) (Figure 1N). Images from the central marginal regions of the specimens were captured at magnifications of 100× and 300×.

### 2.4. Statistical Analysis

Statistical analyses were conducted using GraphPad Prism 8 software (GraphPad Software, San Diego, CA, USA). The mean vertical and horizontal fit values, along with their standard deviations, were calculated for each group. Data were tested for homogeneity, and one-way analysis of variance (ANOVA) followed by the Tukey’s HSD test (α = 0.05), was used to compare the groups. Additionally, the vertical fit was categorized, and a chi-square test (Fisher’s exact) (α = 0.05) was used to analyze the frequencies obtained.

## 3. Results

**Figure 3:**
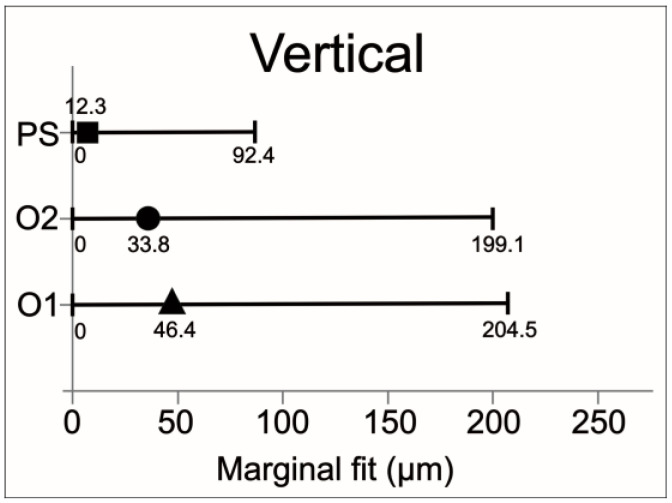
Mean vertical fit values and ranges (µm) of the ceramic crowns according to the experimental groups.

**Figure 4:**
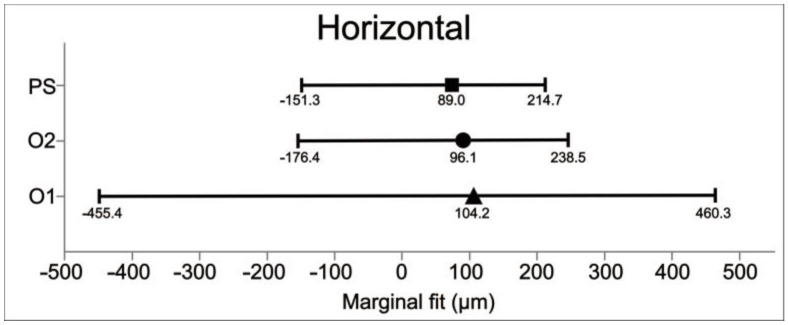
Mean horizontal fit values and ranges (µm) of the ceramic crowns according to the experimental groups.

The mean vertical and horizontal fit values and scanning time for the experimental groups are presented in Table 3. The percentage categories for vertical fit are provided in Table 4. The mean vertical fit values (±SD) were: O1—46.7 ± 16.4 μm; O2—33.8 ± 21.4 μm; and PS—12.3 ± 6.6 μm. For horizontal fit, the mean values (±SD) were: O1—104.2 ± 20.1 μm; O2—96.1 ± 16.9 μm; and PS—89.9 ± 14.2 μm. Figure 3 and Figure 4 illustrate the mean fit values and their ranges for the vertical and horizontal assessments, respectively.

**Figure 5:**
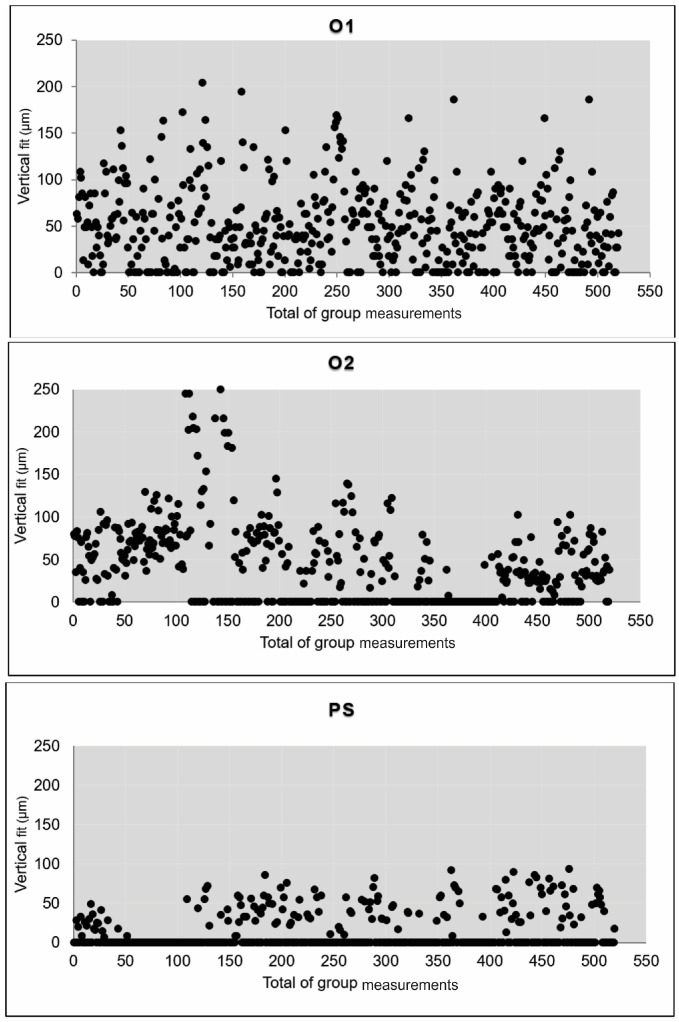
Scatterplots of the vertical fit measurements according to the experimental groups.

**Figure 6:**
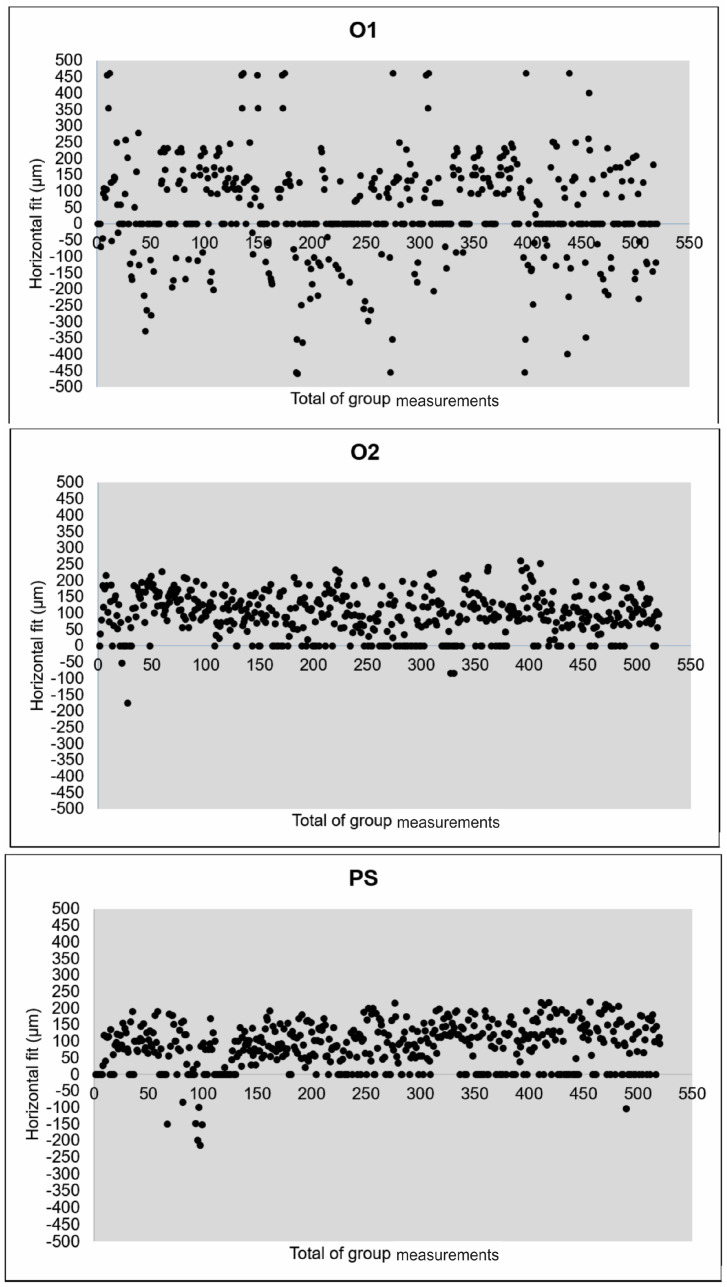
Scatterplots of the marginal horizontal fit measurements according to the experimental groups.

**Figure 7:**
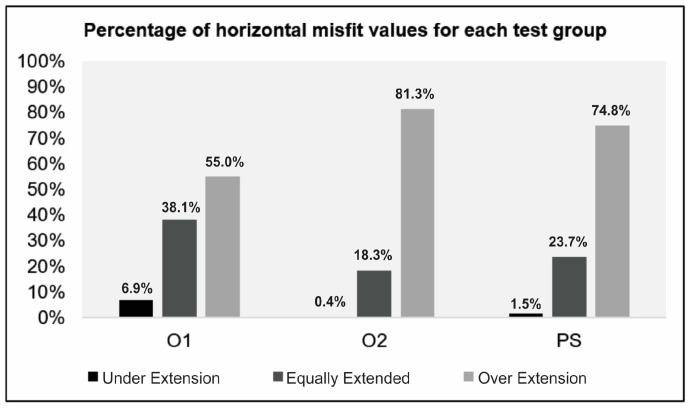
Percentage of horizontal fit values according to the experimental groups.

Considering the vertical fit, the results were significantly different for PS compared to O2 (*p* < 0.0001) and O1 (*p* < 0.0001). O1 also differed from O2 in terms of vertical fit (*p* < 0.0406). Regarding the horizontal fit, there were no significant differences across the groups. In the current study, clinically acceptable values for vertical marginal adaptation were considered up to 120 μm. The percentages of values within this limit and within other limits defined in the literature are shown in Table 4. Considering the scatterplots (Figure 5 and Figure 6), greater variation in vertical and horizontal fit measurements was observed for group O1 (4.2.5 software) when compared to O2 (software 5.0). Figure 7 illustrates which percentages were under-extended, equally extended, and over-extended according to the groups. 

The mean scanning times (±SD) were: O1—37.4 ± 3.1 s; O2—34.8 ± 2.7 s; and PS—27.8 ± 1.9 s and (Table 3). PS showed the shortest scanning time among the IOSs (*p* < 0.0001), significantly differing from O1 and O2, which presented similar scanning times (*p* = 0.054).

**Figure 8:**
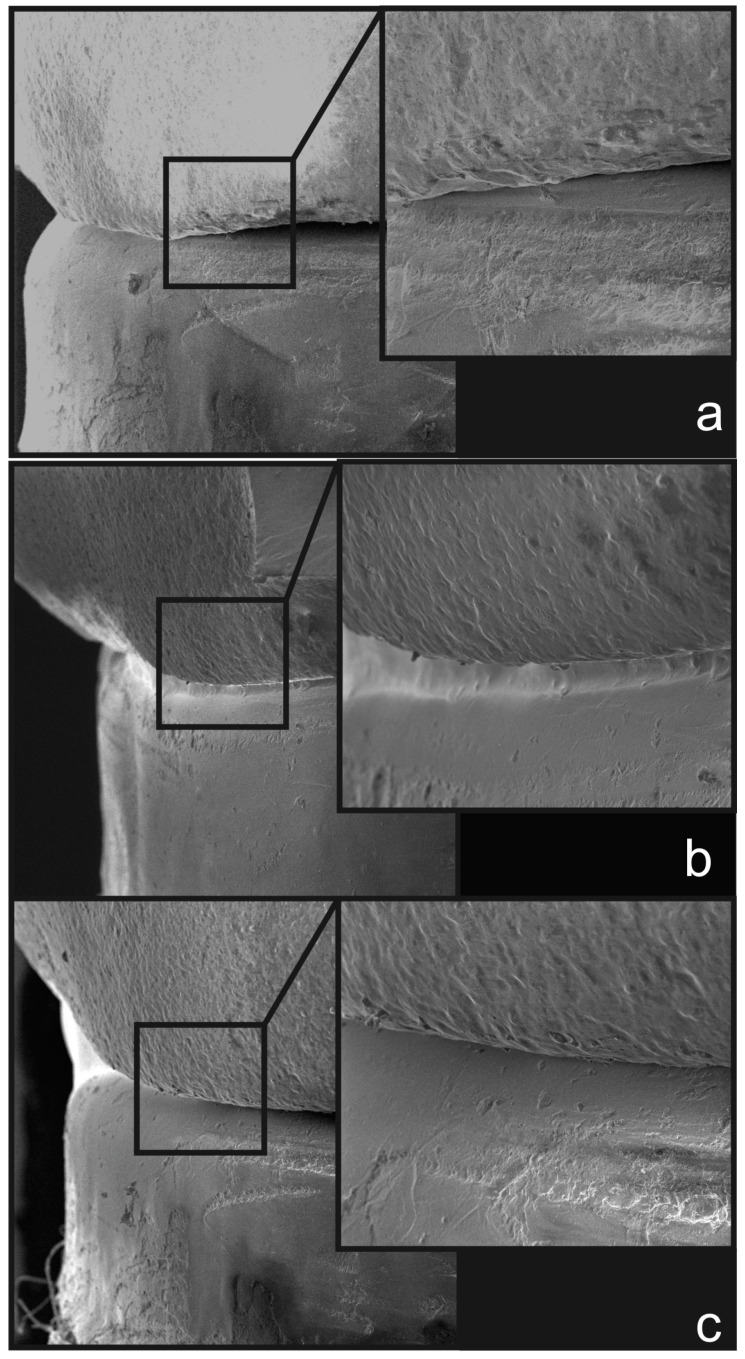
Representative scanning electron microscopy (SEM) images (100× and 300×) of the marginal fit according to the experimental groups: (**a**) O1, (**b**) O2, and (**c**) PS.

Representative SEM images from each experimental group are presented in Figure 8. The O1 group (Figure 8a) exhibited a poor and inconsistent fit, as confirmed by the scatterplots. In contrast, group O2 (Figure 8b) showed a moderate fit, while the PS group (Figure 8c) displayed the best fit.

## 4. Discussion

Since the scanning time and marginal fit of ceramic crowns produced with the evaluated intraoral optical scanner (IOS) technologies differed among the systems, the proposed null hypothesis was rejected. New technologies tend to present better results [16], and the camera hardware and software of the PS system offered fast scanning times and superior vertical marginal fit for ceramic crowns produced with the same milling unit, compared to the other IOS systems in this study. The blue-light and confocal microscopy technology present in the PS system, along with improved software (CEREC 3D v. 5.0), enhanced the 3D model acquisition and crown design, optimizing both the milling step and marginal fit of the ceramic crowns. Moreover, the PS system can easily detect sharp areas in the image to calculate the distance to the object, which corresponds to the focal length of the lens [15]. These factors may explain why 98.1% of the crowns produced with the PS system exhibited vertical marginal fit values below 75 µm [10,30], and 81.6% fell below 30 µm—considered as the gold standard values.

Marginal fit is critical for the clinical success of indirect restorations [8,17]. Well-fitting restorations minimize risks, such as microleakage, plaque accumulation, caries, periodontal inflammation, and endodontic lesions, which influence the clinical longevity of single-crown rehabilitations [8,9,17,18,19]. Research has shown that hardware and software updates in IOS systems improve marginal fit [3,6,10,16]. Comparing the same IOS hardware with upgraded software (O1 vs. O2) and updated computer hardware, the improved scanning technology of the O2 system resulted in superior vertical marginal fit using CEREC 3D v. 5.0 than the O1 system with CEREC v. 4.2.5.

The findings of the current study highlight that both hardware and software upgrades enhance restoration fit, in addition to improving digital models [5]. The improved marginal detection capabilities of the new software (CEREC 3D v.5.0) compared to the earlier version (CEREC v.4.2.5) and the greater precision achieved with software advancements justify these outcomes. Haddad et al. (2018), found that updated software versions yielded better crown adaptation values [45]. Additionally, newer software versions reduced scanning times, while improving accuracy through enhanced image capture [46]. Older software (v. 4.2.5) likely generated more reconstruction errors, producing thinner margin demarcation lines, that impaired margin delineation and adversely impacted the O1 vertical marginal fit. Surface irregularities in the digital models of the O1 group may also have affected crown dimensions, resulting in the highest vertical marginal discrepancies.

The current study evaluated the marginal fit of CAD/CAM-manufactured LS2 crowns produced with different IOS systems using micro-CT, a non-destructive method that enables the assessment of marginal and internal fit without altering the marginal region [39,40,41]. In a prior investigation, the Primescan group outperformed the Omnicam group in scan trueness and precision [7]. Micro-CT analysis enabled evaluation of crown fit across various areas of tooth preparation. However, this method depends on operator calibration for accurate measurements [47], necessitating the application of Cohen’s kappa coefficient for intra-rater reliability [10]. Handling and configuring the equipment also poses technical challenges [47], which were mitigated in the current study by following protocols from previous research [9,10,15].

This study categorized the vertical fit into ranges: less than 10 µm, 10.01 to 30 µm [31], 30.01 to 75 µm [30,31], 75.01 to 120 µm [24], and greater than 120 µm [24]. These categories were based on previous studies defining clinically acceptable fit values and the pixel size used. Marginal fit within 10 μm is considered clinically negligible, even with 9.4 μm variability. Thus, this pixel size does not affect measurement consistency, as margins exceeding 120 µm are detectable with an explorer [23]. The pixel size of 9.4 μm was a limitation of this study in detecting marginal fits below this value. Although pixel size limitations restricted detection of fits under 9.4 µm, values exceeding 9.4 µm were reliably measured. Fits below 25–40 μm were rare and considered excellent [3,6,10,16].

Scanning time is essential for patient comfort [14], and advancements in hardware and software technologies have significantly reduced the chairside time. Shorter scanning duration minimizes the influence of crevicular fluid, ambient lighting, breathing, saliva, patient movements, and restricted access, improving the quality of 3D models and restorative fit. Operator experience also enhances the scanning accuracy, with clinical practice reducing deviations and improving scan precision [20]. The current study standardized conditions by scanning a typodont model, eliminating intraoral variables.

According to ISO 5625:1978 [47] “precision” refers to the closeness of the results obtained under standardized conditions, expressed through standard deviation [15]. In the current study, the PS group showed lower SDs, indicating higher precision. This was further supported by the scatterplots, which demonstrated less variability in the measurements from the PS group. Thus, the PS group exhibited the highest IOS accuracy, followed by the O2 and O1, likely due to the newer image capture technologies and software used.

This study evaluated two versions of the Omnicam system and the Primescan system, using video camera technology [5]. However, the PS group utilized blue light (present in the Bluecam system) and confocal technology, while the O1 and O2 systems employed white light and triangulation [15]. This shift to blue light technology, with its shorter and more intense wavelength, may explain the superior performance of the PS system [9]. Previous studies comparing Omnicam with Bluecam found better crown adaptation with Bluecam technology [25]. Although video scanning is faster than image capture, the superior results of the PS group suggest that combining blue light with confocal technology plays a significant role in performance.

Several methods have been used to assess the marginal adaptation of indirect restorations, including stereomicroscopy, scanning electron microscopy [33,34,35], optical microscopy, and micro-CT [9,32,36,37,38,39,40]. Micro-CT has emerged as one of the most effective methods due to its high-resolution, non-destructive 3D analysis, and ability to perform repeated measurements [9,10,15,32,36,37,38,39,40]. Other methods offer 2D analysis using microscopy combined with silicone replica techniques [48], facilitating measurement acquisition. Regardless of the method used, a minimum of 50 measurement points per crown is recommended to ensure reliable adaptation estimates [49].

The current in vitro study presents inherent limitations. Factors such as subgingival margins, patient movements, saliva, and lighting can influence outcomes [5]. These variables were controlled in the lab, but clinical studies are needed to confirm these findings. The composite resin die used simulated coronal restorations for valid IOS comparisons. Future studies should assess how internal adjustments affect marginal fit with other IOS systems. Micro-CT settings, including pixel size and reconstruction parameters, could affect image quality and measurement accuracy, posing additional challenges.

## 5. Conclusions

Within the limitations of this in vitro study, the following conclusions were drawn: (1) the Primescan system provided superior marginal fit for ceramic crowns and shorter scanning time compared to Omnicam systems; and (2) hardware and software improvements in IOS systems significantly influenced the marginal fit of ceramic crowns produced via CAD/CAM technology.

## Figures and Tables

**Table 1:** Description of experimental groups (n = 10).

IOS System	Software	Milling Unit	Groups	Ceramic
Omnicam 1.0	v4.2.5	MCXL	O1	Lithium disilicate
Omnicam 2.0	v5.0	MCXL	O2	Lithium disilicate
Primescan	v5.0	MCXL	PS	Lithium disilicate

**Table 2:** Crystallization process.

Furnace	Closing Time (min)	Stand-By: Temperature (°C/°F)	Heating Rate: t_1_ °C/min/°F/min	Fitting Temperature: T_1_ °C/°F	Holding Time: H_1_ min	Heating Rate: T_2_ °C/min/°F/min	Fitting Temperature: T_2_ °C/°F	Holding Time: H_2_ min	Long-term Cooling: L °C/min/°F/min	Cooling Rate: t_1_ °C/min/°F/min	Vacuum 1: 1_1_ 1_1_	Vacuum 2: 2_1_ 2_2_
P300	6:00	403/757	60/108	770/1418	5:00	30/54	850/1562	10:00	700/1292	20/36	550/1022 770/1418	770/1418 850/1562

**Table 3:** Mean (±SD) vertical and horizontal marginal fit (µm) and scanning time (s) values according to the experimental groups (*n* = 10).

IOS System	Vertical (µm)	Horizontal (µm)	Scanning Time (s)
O1	46.7 ± 16.4 ^c^	104.2 ± 20.1 ^b^	37.4. ± 3.1 ^C^
O2	33.8 ± 21.4 ^b^	96.1 ± 16.9 ^a^	34.8 ± 2.7 ^B^
PS	12.3 ± 6.6 ^a^	89.0 ± 14.2 ^a^	27.8 ± 1.9 ^A^

Values with the same superscript letter are not significantly different (columns) based on the Tukey’s HSD test (*p* < 0.05).

**Table 4:** Vertical fit percentage (%) categories according to the experimental groups (*n* = 10).

IOS System	≥10 µm	10.01 to 30 µm	30.01 to 75 µm	75.01 to 120 µm	≤120 µm
O1 ^c^	23.5%	12.3%	43.7%	16.2%	4.3%
O2 ^b^	35.4%	6.2%	26.3%	14.6%	17.5%
PS ^a^	72.3%	5.4%	18.7%	3.7%	0.0%

Values with the same superscript letter are not significantly different based on the chi-square test (Fisher’s exact) (*p* < 0.05).

## Data Availability

The original contributions presented in the study are included in the article, and further inquiries can be directed to the corresponding author.

## References

[1] Anadioti E., Aquilino S.A., Gratton D.G., Holloway J.A., Denry I., Thomas G.W., Quian F. (2014). 3D and 2D Marginal Fit of Pressed and CAD/CAM Lithium Disilicate Crowns Made from Digital and Conventional Impressions: Marginal Fit of All-Ceramic Crowns. J. Prosthodont..

[2] Kocaağaoğlu H., Albayrak H., Cinel Sahin S., Gürbulak A.G. (2019). Evaluation of marginal adaptation in three-unit frameworks fabricated with conventional and powder-free digital impression techniques. J. Adv. Prosthodont..

[3] Ender A., Zimmermann M., Attin T., Mehl A. (2016). In vivo precision of conventional and digital methods for obtaining quadrant dental impressions. Clin. Oral Investig..

[4] Zimmermann M., Ender A., Mehl A. (2020). Local accuracy of actual intraoral scanning systems for single-tooth preparations in vitro. J. Am. Dent. Assoc..

[5] Abduo J., Elseyoufi M. (2018). Accuracy of Intraoral Scanners: A Systematic Review of Influencing Factors. Eur. J. Prosthodont. Restor. Dent..

[6] Ender A., Zimmermann M., Mehl A. (2019). Accuracy of complete- and partial-arch impressions of actual intraoral scanning systems in vitro. Int. J. Comput. Dent..

[7] Zimmermann M., Mehl A., Mörmann W.H., Reich S. (2015). Intraoral scanning systems—A current overview. Int. J. Comput. Dent..

[8] Akat B., Şentürk A., Ocak M., Kiliçarslan M.A., Özcan M. (2022). Does cad software affect the marginal and internal fit of milled full ceramic crowns?. Braz. Oral Res..

[9] Neves F.D., Prado C.J., Prudente M.S., Carneiro T.A.P.N., Zancopé K., Davi L.R., Mendonça G., Cooper L., Soares C.J. (2014). Micro-computed tomography evaluation of marginal fit of lithium disilicate crowns fabricated by using chairside CAD/CAM systems or the heat-pressing technique. J. Prosthet. Dent..

[10] Prudente M.S., Davi L.R., Nabbout K.O., Prado C.J., Pereira L.M., Zancopé K., Neves F.D. (2018). Influence of scanner, powder application, and adjustments on CAD-CAM crown fit. J. Prosthet. Dent..

[11] Zingari F., Meglioli M., Gallo F., Macaluso G.M., Tagliaferri S., Toffoli A., Ghezzi B., Lumetti S. (2023). Predictability of intraoral scanner error for full-arch implant-supported rehabilitation. Clin. Oral Investig..

[12] Wesemann C., Kienbaum H., Thun M., Spies B.C., Beuer F., Bumann A. (2021). Does ambient light affect the accuracy and scanning time of intraoral scans?. J. Prosthet. Dent..

[13] Logozzo S., Zanetti E.M., Franceschini G., Kilpelä A., Mäkynen A. (2014). Recent advances in dental optics—Part I: 3D intraoral scanners for restorative dentistry. Opt. Lasers Eng..

[14] Richert R., Goujat A., Venet L., Viguie G., Viennot S., Robinson P., Farges J., Fages M., Ducret M. (2017). Intraoral Scanner Technologies: A Review to Make a Successful Impression. J. Healthc. Eng..

[15] Kim R.J.Y., Benic G.I., Park J.M. (2021). Trueness of ten intraoral scanners in determining the positions of simulated implant scan bodies. Sci. Rep..

[16] Mehl A., Ender A., Mörmann W., Attin T. (2009). Accuracy testing of a new intraoral 3D camera. Int. J. Comput. Dent..

[17] Baig M.R., Tan K.B.C., Nicholls J.I. (2010). Evaluation of the marginal fit of a zirconia ceramic computer-aided machined (CAM) crown system. J. Prosthet. Dent..

[18] Pak H.S., Han J.S., Lee J.B., Kim S.H., Yang J.H. (2010). Influence of porcelain veneering on the marginal fit of Digident and Lava CAD/CAM zirconia ceramic crowns. J. Adv. Prosthodont..

[19] Sorensen J.A. (1989). A rationale for comparison of plaque-retaining properties of crown systems. J. Prosthet. Dent..

[20] Lim J.H., Park J.M., Kim M., Heo S.J., Myung J.Y. (2018). Comparison of digital intraoral scanner reproducibility and image trueness considering repetitive experience. J. Prosthet. Dent..

[21] Ates S.M., Yesil Duymus Z. (2016). Influence of Tooth Preparation Design on Fitting Accuracy of CAD-CAM Based Restorations. J. Esthet. Restor. Dent..

[22] Goodacre C.J., Campagni W.V., Aquilino A.S. (2001). Tooth preparations for complete crowns: An art form based on scientific principles. J. Prosthet. Dent..

[23] Colpani J.T., Borba M., Della Bona A. (2013). Evaluation of marginal and internal fit of ceramic crown copings. J. Prosthet. Dent..

[24] McLean J.W., Von F. (1971). The estimation of cement film thickness by an in vivo technique. Braz. Dent. J..

[25] das Neves F.D., de Almeida Prado Naves Carneiro T., do Prado C.J., Prudente M.S., Zancopé K., Davi L.R., Mendonça G., Soares C.J. (2014). Micrometric precision of prosthetic dental crowns obtained by optical scanning and computer-aided designing/computer-aided manufacturing system. J. Biomed. Opt..

[26] Beschnidt S.M., Strub J.R. (1999). Evaluation of the marginal accuracy of different all-ceramic crown systems after simulation in the artificial mouth. J. Oral Rehabil..

[27] Dolev E., Bitterman Y., Meirowitz A. (2019). Comparison of marginal fit between CAD-CAM and hot-press lithium disilicate crowns. J. Prosthet. Dent..

[28] Davis D.R. (1988). Comparison of fit of two types of all-ceramic crowns. J. Prosthet. Dent..

[29] Keshvad A., Hooshmand T., Asefzadeh F., Khalilinejad F., Alihemmati M., Van Noort R. (2011). Marginal Gap, Internal Fit, and Fracture Load of Leucite-Reinforced Ceramic Inlays Fabricated by CEREC inLab and Hot-Pressed Techniques: Marginal Adaptation of Machined and Pressed Leucite Ceramic. Inlays J. Prosthodont..

[30] Hung S.H., Hung K.S., Eick J.D., Chappell R.P. (1990). Marginal fit of porcelain-fused-to-metal and two types of ceramic crown. J. Prosthet. Dent..

[31] Council Adopts American Dental Association Specification No. 8 (1967). Dental Zinc Phosphate Cement and 11 Agar Impression Material. J. Am. Dent. Assoc..

[32] May K.B., Russell M.M., Razzoog M.E., Lang B.R. (1998). Precision of fit: The Procera AllCeram crown. J. Prosthet. Dent..

[33] Bindl A., Mörmann W.H. (2005). Marginal and internal fit of all-ceramic CAD/CAM crown-copings on chamfer preparations. J. Oral Rehabil..

[34] Vanlioğlu B.A., Evren B., Yildiz C., Uludamar A., Özkan Y.K. (2012). Internal and marginal adaptation of pressable and computer-aided design/computer-assisted manufacture onlay restorations. Int. J. Prosthodont..

[35] Trifkovic B., Budak I., Todorovic A., Hodolic J., Puskar T., Jevremovic D., Vukelic D. (2012). Application of replica technique and SEM in accuracy measurement of ceramic crowns. Meas. Sci. Rev..

[36] Coli P., Karlsson S. (2004). Fit of a New Pressure-Sintered Zirconium Dioxide Coping. Int. J. Prosthodont..

[37] Pelekanos S., Koumanou M., Koutayas S.O., Zinelis S., Eliades G. (2009). Micro-CT evaluation of the marginal fit of different In-Ceram alumina copings. Eur. J. Esthet. Dent. Off. J. Eur. Acad. Esthet. Dent..

[38] Di Fiore A., Zuccon A., Carraro F., Basilicata M., Bollero P., Bruno G., Stellini E. (2023). Assessment Methods for Marginal and Internal Fit of Partial Crown Restorations: A Systematic Review. J. Clin. Med..

[39] Attia M.A., Blunt L., Bills P., Tawfik A., Radawn M. (2023). Micro-CT analysis of marginal and internal fit of milled and pressed polyetheretherketone single crowns. J. Prosthet. Dent..

[40] Toma F.R., Moleriu L.C., Porojan L. (2023). Micro-CT Marginal and Internal Fit Evaluation of CAD/CAM High-Performance Polymer Onlay Restorations. Polymers.

[41] Demir N., Ozturk A.N., Malkoc M.A. (2014). Evaluation of the marginal fit of full ceramic crowns by the microcomputed tomography (micro-CT) technique. Eur. J. Dent..

[42] Ekici Z., Kılıçarslan M.A., Bilecenoğlu B., Ocak M. (2021). Micro-CT Evaluation of the Marginal and Internal Fit of Crown and Inlay Restorations Fabricated Via Different Digital Scanners belonging to the Same CAD-CAM System. Int. J. Prosthodont..

[43] Osman R.B., Alharbi N.M. (2023). Influence of scan technology on the accuracy and speed of intraoral scanning systems for the edentulous maxilla: An in vitro study. J. Prosthodont..

[44] Raposo L.H., Borella P.S., Ferraz D.C., Pereira L.M., Prudente M.S., Santos-Filho P.C. (2020). Influence of Computer-aided Design/Computer-aided Manufacturing Diamond Bur Wear on Marginal Fit of Two Lithium Disilicate Ceramic Systems. Oper. Dent..

[45] Haddadi Y., Bahrami G., Isidor F. (2018). Effect of Software Version on the Accuracy of an Intraoral Scanning Device. Int. J. Prosthodont..

[46] Porr D.A., Brooks D.I., Liacouras P.C., Petrich A., Ellert D.O., Ye L. (2022). Time and Accuracy of the CEREC Omnicam Using Two Different Software Programs. J. Prosthodont..

[47] (1978). Shipbuilding—Welded bulkhead pieces with flanges for steel pipework—PN 6, PN 10 and PN 16.

[48] Groten M., Axmann D., Pröbster L., Weber H. (2000). Determination of the minimum number of marginal gap measurements required for practical in-vitro testing. J. Prosthet. Dent..

[49] Zanini F., Carmignato S., Gao W. (2019). X-Ray Computed Tomography for Dimensional Metrology. Metrology. Precision Manufacturing.

